# Supplementary material for: A network dynamical simulation model for the study of antibiotic resistance in microbial communities
Source: Gut Microbes. 2026 Sep 19;18(1):2734703. doi: 10.1080/19490976.2026.2734703 (PMC13596919; doi:10.1080/19490976.2026.2734703)
Supplement: Supplementary file 1 [file KGMI_A_2734703_SM9053.zip › supplementary_file_1/Figures/ba_delta_mean_distance_gc_n300.pdf]

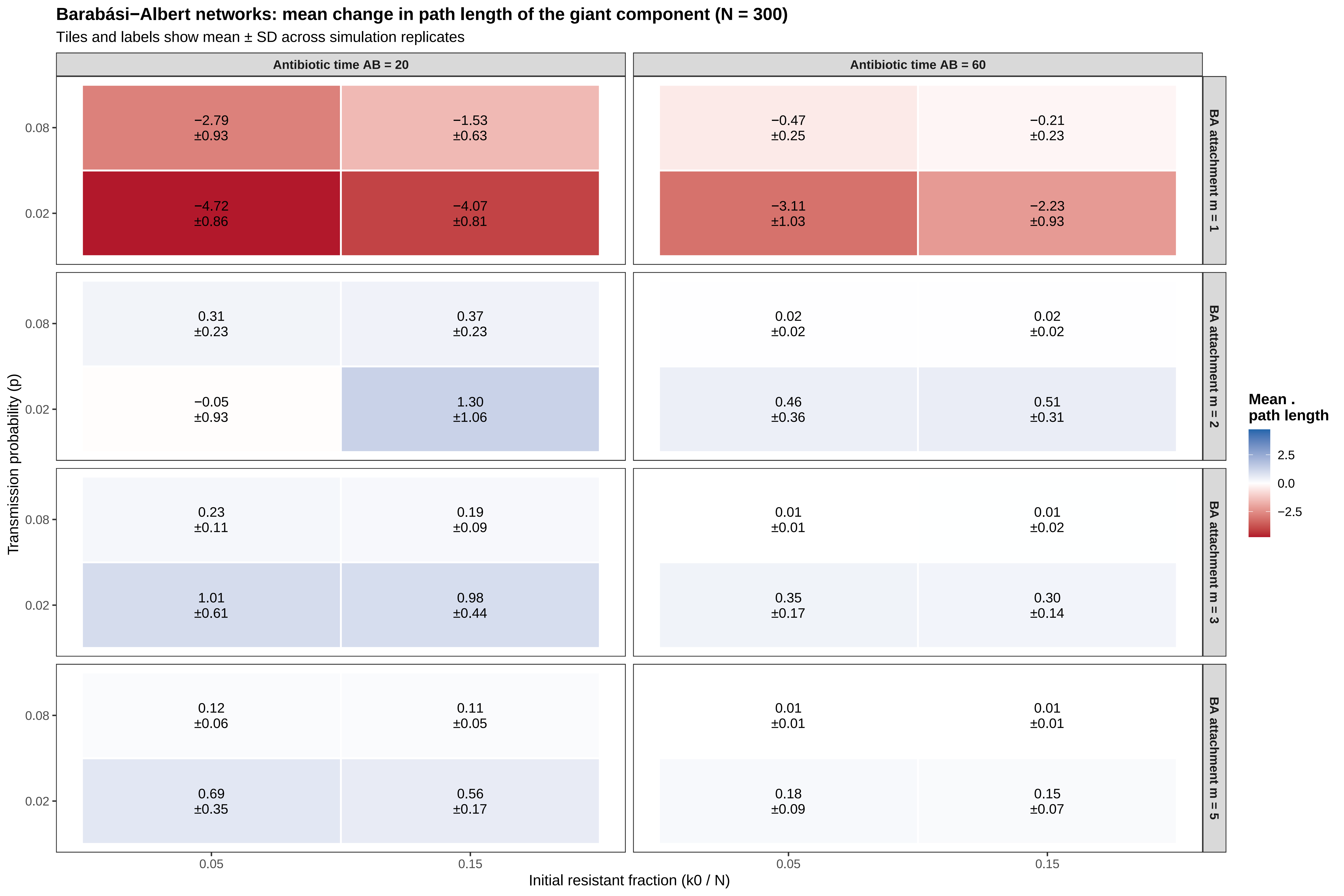

Barabási−Albert networks: mean change in path length of the giant component (N = 300)
Tiles and labels show mean ± SD across simulation replicates
Antibiotic time AB = 20
Antibiotic time AB = 60
BA attachment m = 1
BA attachment m = 2
BA attachment m = 3
BA attachment m = 5
−2.79 ±0.93
−1.53 ±0.63
−4.72 ±0.86
−4.07 ±0.81
−0.47 ±0.25
−0.21 ±0.23
−3.11 ±1.03
−2.23 ±0.93
0.31 ±0.23
0.37 ±0.23
−0.05 ±0.93
1.30 ±1.06
0.02 ±0.02
0.02 ±0.02
0.46 ±0.36
0.51 ±0.31
0.23 ±0.11
0.19 ±0.09
1.01 ±0.61
0.98 ±0.44
0.01 ±0.01
0.01 ±0.02
0.35 ±0.17
0.30 ±0.14
0.12 ±0.06
0.11 ±0.05
0.69 ±0.35
0.56 ±0.17
0.01 ±0.01
0.01 ±0.01
0.18 ±0.09
0.15 ±0.07
Transmission probability (p)
0.08
0.02
Initial resistant fraction (k0 / N)
0.05
0.15
Mean .
path length
2.5
0.0
−2.5
